# Klinefelter syndrome in a Holstein-Friesian bull: a case report

**DOI:** 10.1186/s13620-026-00346-2

**Published:** 2026-04-25

**Authors:** ET Kelly, D Sheehan, JP Cassidy, CM O Meara, D Corridan, CA Ryan, DP Berry, A Collins, CC Rathje, ME Beltman

**Affiliations:** 1 UCD Veterinary Hospital, UCD School of Veterinary Medicine, Belfield, Dublin 4, D04W6F6 Ireland; 2 Teagasc, Moorepark Dairy Production Research Centre, Co. Cork P61C996 Fermoy, Ireland; 3 National Cattle Breeding Centre, Unit K4, M7 Business Park, Co. Kildare, W91WF59 Ireland; 4 Kent University, Canterbury, CT2 7NU UK

## Abstract

**Background:**

This report describes the clinical, laboratory, and post-mortem findings in a 16-month-old Holstein-Friesian bull diagnosed with Klinefelter syndrome, a rare sex chromosome disorder characterised by testicular hypoplasia and infertility.

**Case presentation:**

During the routine genotype quality control process, the bull was flagged as a suspected XXY (Klinefelter syndrome) based on its genotype data. Analysis of parental genotype data revealed the bull inherited one X chromosome from each parent, indicating a paternal nondisjunction event. Karyotyping subsequently confirmed the 61,XXY chromosome complement. Clinical examination revealed the bull had normal libido with normal testosterone concentration (13.1 nmol/ml) but had a small scrotal circumference (18.5 cm). Post-mortem examination revealed that the bull had bilateral testicular hypoplasia and azoospermia. These findings were consistent with other reports of Klinefelter syndrome in bulls.

**Conclusion:**

This case highlights the importance of the national genotyping screening program to identify infertile bulls (and females) that should not be considered for breeding. The clinical and post-mortem observations add to the limited veterinary literature on bovine Klinefelter syndrome presentations.

## Background

Klinefelter syndrome is a sex chromosome aneuploidy characterised by the presence of at least one supernumerary X chromosome, resulting in a 47,XXY karyotype in the majority of cases. With a prevalence of approximately 1 in 600 male births in humans [1], it is the most common genetic cause of male hypogonadism and is associated with infertility, testicular hypoplasia, and azoospermia. In animals, the most common phenotypic representation of Klinefelter syndrome is in male tortoiseshell and calico cats [2] which have a characteristic tri-colour coat. Klinefelter syndrome has also been documented in cattle [3] horses [4], and sheep [5], where affected males consistently present with infertility, testicular hypoplasia, and azoospermia despite a normal male phenotype in the majority of cases. The diagnosis is often retrospective, made during investigations of infertility [6, 7].

Routine aneuploidy screening using readily available single nucleotide polymorphism (SNP) genotype data can identify conditions such as sex chromosome monosomy (e.g., XO) or trisomy (e.g., XXY) prior to breeding [8, 9]. There is limited information regarding Klinefelter syndrome in bovines. Logue at al. reported the syndrome in a XXY Friesian bull that had normal libido with bilateral testicular hypoplasia, azoospermia, smaller and shorter seminiferous tubules and relative prominence of Leydig cells [3]. Dunn et al. reported a Hereford bull with testicular hypoplasia and azoospermia. The Hereford bull’s testes weighed approximately 10% of the normal testicular weight of age and breed-matched controls [7]. Schmutz et al. [6] reported the syndrome in a Charolais bull that was smaller in stature and had smaller testes than its herd mates and produced an azoospermic ejaculate. This animal had normal libido and had normally developed accessory sex glands suggesting normal testosterone production. Histological examination of the animal’s testes post castration revealed normal numbers of Leydig cells but few Sertoli cells.

The case presented here describes a karyotypic diagnosis of Klinefelter syndrome in a 16-month-old Holstein-Friesian bull, with the subsequent clinical, endocrine and histological findings.

## Case presentation

### Signalment and history

The National Cattle Breeding Centre (NCBC) regularly contracts bull calves for its genomic breeding programs. A young dairy bull calf was contracted to the stud by the breeding program manager in January 2024. The bull was retained in the rearing units until December 2024. At that stage the bull was moved to the pre-quarantine facilities for handling and training ahead of moving to the main barns to enter one of the semen collection units.

The bull underwent teasing and semen collection for exploratory purposes and was found to have normal libido and willing to mount a teaser for ejaculation into an artificial vagina. He was managed under standard husbandry conditions for artificial insemination (AI) stud practice (including blood sampling for diseases under EU Directive 88/407) within calf rearing units and quarantine stud facilities. The semen of the bull was collected for research purposes only and 7 ejaculates were assessed for volume and sperm cell concentration. Ejaculate volumes were normal (range 1-3 ml). Colour was normal but appeared watery and not dense. Concentration was assessed initially by flow cytometry (Cytoflex, Becman Coulter) and subsequently by New improved Neubauer hemocytometer (0.0025mm2; Brand, Germany) on the raw semen ejaculate and subsequently confirmed using hemoctyometer. Concentrations were low using flow cytometry (<10 x 10 6 /ml) and this was confirmed via hemoctyometer. Motility was assessed by removing an aliquot of semen to a glass slide and covered with a coverslip maintained at 37֩C on a heated stage. The semen sample was examined under a phase contrast microscope (x10 and x20 magnification; D2500, Leica, Germany) for the presence of spermatozoa. The ejaculates were allowed to settle, and the remaining pellet was used for morphological assessments. Smears of the semen samples were fixed on glass slides and stained using a Spermac staining kit for morphological assessment as per manufacturer’s instructions ( Minitube, Germany). Irregular cells were noted at extremely low levels (in the order of 1 cell per 1 ml of ejaculate). There was no presence of morphologically normal spermatozoan noted following staining. The XXY bull (aged 486 days old) appeared smaller in stature with smaller testicular dimensions than calves of similar age but otherwise presented as clinically normal before being transported to the University College Dublin Veterinary Hospital for further examination, euthanasia and post-mortem examination.

### Clinical and reproductive examination

**Fig. 1:**
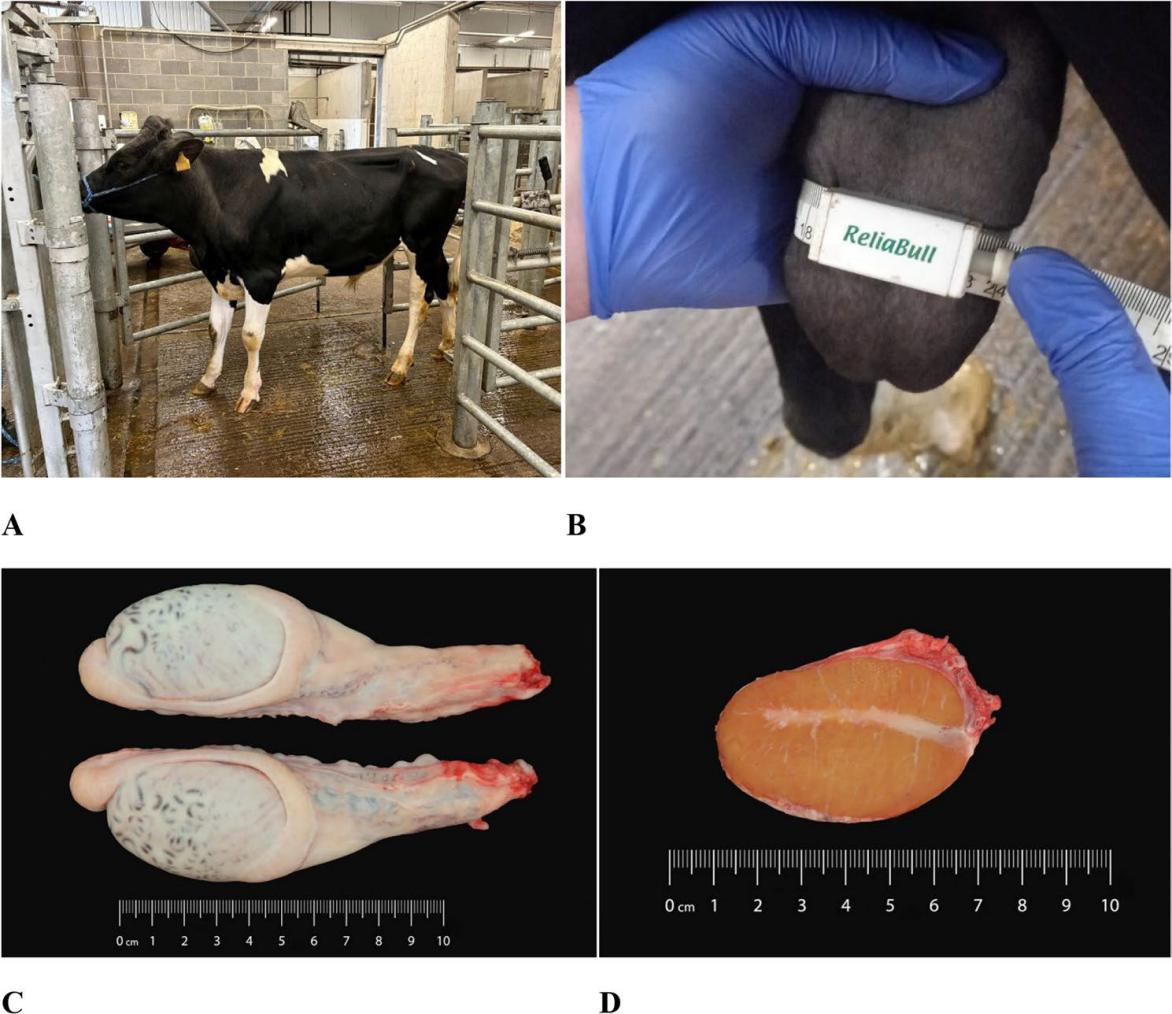
Ante- and post-mortem images of the Holstein-Friesian bull and his testes. **A** Bull was 16 months of age and was smaller in stature (380 kg) versus age matched controls with a (**B**) markedly reduced scrotal circumference of 18.5 cm (ReliaBull ©). **C** Gross appearance of both testes and (**D**) cross section of the right testes appeared normal. However, the testes were significantly reduced in size and weight (left = 45 g & right = 44 g) indicating a testicular hypoplasia

Ethical exemption was obtained for all ante- and post-mortem examinations of the bull (AREC-E-25-32-Beltman) in the University College Dublin Veterinary Hospital. On admission, the bull weighed 380 kg and measured 135 cm at the withers (Fig. 1A). Clinical examination revealed no abnormal clinical parameters other than that bilaterally the testes were appeared very small for his age (Fig. 1B) and the bull had a total scrotal circumference at its widest point of 18.5 cm when measured with a measuring tape (ReliaBull ^©^). Testes were of normal consistency (i.e., firm but not hard) on palpation and freely movable in the scrotum. Ultrasonographic examination of the external and internal genitalia was performed using an Easi Scan Go ultrasound scanner (IMV Imaging) and revealed both testes and epididymi to be normal in structure and anatomical position, with a clear distinction between the testicular parenchyma and the mediastinum. The epididymis both had a caput and cauda which appeared more hypoechoic than the testicular structure, consistent with the appearance found in a normal animal. The prostate, ampullae and seminal vesicles palpated normally on rectal examination and were of normal appearances on rectal ultrasonographic examination, with the vesicles each appearing as a large lobulated structure that was heterogenous, the ampullae appearing thick walled with some fluid present and the prostate appearing as a band shaped structure directly on top of the pelvic urethra.

### Blood sampling

A jugular venous 12G short stay catheter (KRUUSE Large Animal IV Catheter) was placed following routine skin preparation with chlorhexidine and alcohol, after which blood samples were taken for routine haematology and biochemistry as well as a single testosterone measurement. Blood sampling through the catheter was performed in the morning at approximately 9 am. The sample was spun down and its testosterone concentration was measured by ELISA post-ether extraction (Nationwide Laboratory Specialists, UK). The testosterone concentration was as 13.1 nmol/ml which was within the dynamic range of the assay (0.03-20.0 nmol/ml) indicating normal concentrations of testosterone [10].

Routine haematology and biochemistry analysis revealed a mild decreased haematocrit concentration (0.25 L/L; ref 0.28–0.38) with a hypoproteinaemia (64.3 g/l; ref 67–85) driven by a mild hypoglobulinaemia (28.50 g/l; ref 31–55) which may have been the result of a mild non-specific immunosuppression or artefact. An elevated creatine kinase (180 U/L; ref < 36 U/L) was likely due to recent transport to the University College Dublin Veterinary Hospital and a mild hyperphosphataemia (2.6 mmol/l; ref 1.2–2.3) and hyperglycaemia (4.11 mmol/l; ref 2.8–3.8) may have been due to iatrogenic haemolysis during sampling and stress, respectively. Lastly, there was a mild neutropenia present with a reduced segmented count of 0.81 × 10⁹/L and differential of 14% (ref 1.16–6.4 and 15–45%) indicating the possibility of the the beginning of an acute inflammatory process.

### Post-mortem and histopathological examinations

**Fig. 2:**
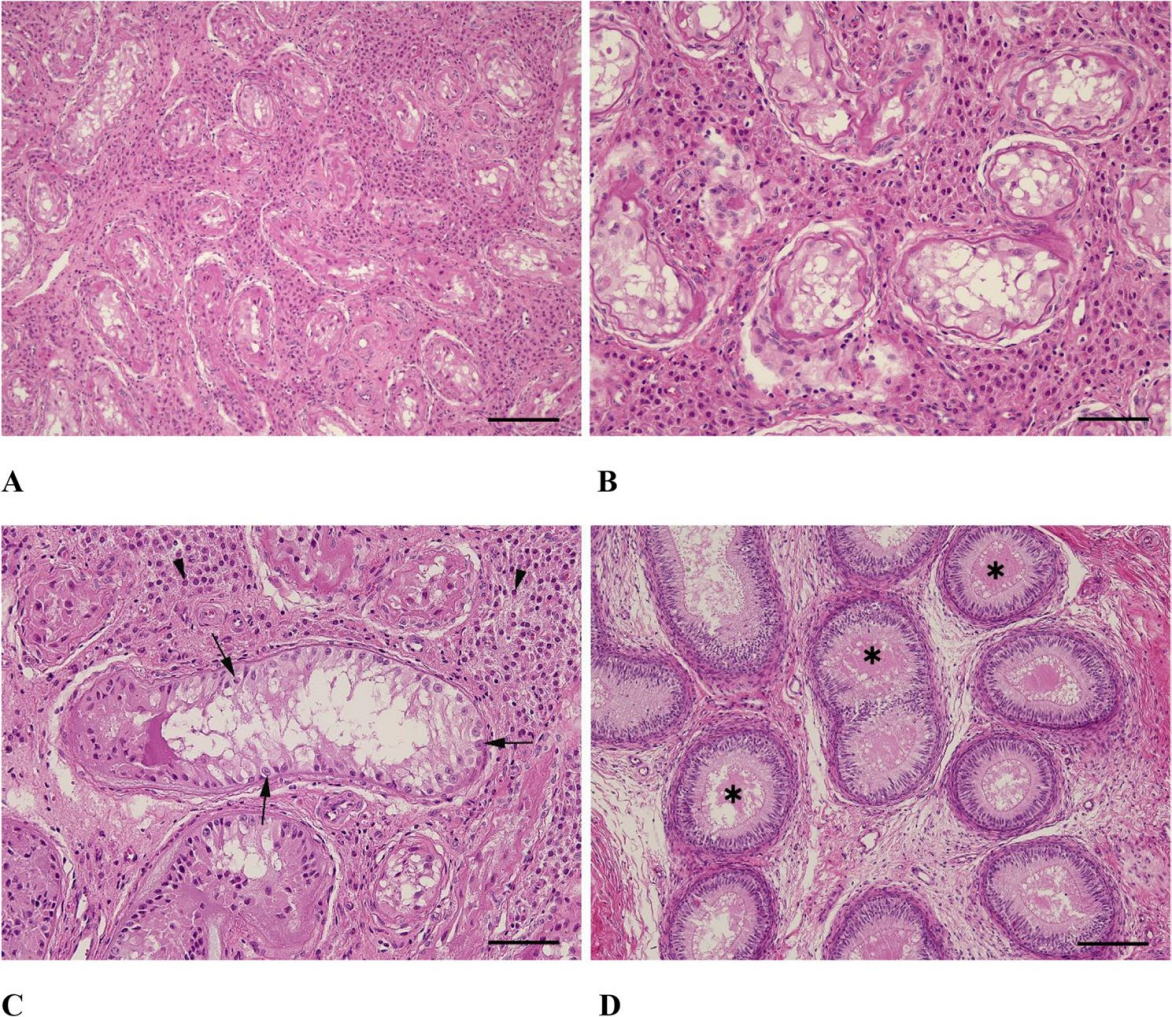
Histology of the testicular tissues. **A**,** B** and** C.** Hypoplastic seminiferous tubules of reduced diameter with no spermatogenesis evident. Only Sertoli cells are visible within the tubulus (C, arrows) and Leydig cells dominate the inter-tubular interstitium (arrowheads). H&E Stain, scale bar A = 250 μm, B = 100 μm, C = 50 μm. **D.** Epididymis with absence of luminal spermatozoa (stars). H&E Stain, scale bar = 250 μm

Abnormal findings post-mortem were restricted to just the reproductive tract. Total penile length was 33 cm from the tip of the glans to the preputial orifice, extended. The free portion of the penis was 11 cm long. In its natural (non-extended) position, the penis measured approximately 25 cm in length. Both the accessory sex glands and testes appeared grossly structurally normal. The combined weight of the accessory sex glands were: vesicular glands 32 g, the ampullae 11 g, and bulbourethral glands 7 g. The left testicle weighed 45 g (Fig. 1C) and was kept intact in formalin for any potential future analysis. The right testicle weighed 44 g (Fig. 1C) and measured 4 cm × 6 cm × 3 cm in size (Fig. 1D). The right epididymis when removed from the testis proper weighed 10 g. Samples of vesicular glands, ampullae, bulbourethral glands, ductus deferens and of right testis and epididymis were taken for histopathological examination. Vesicular glands, ampullae, and bulbourethral glands appeared normal microscopically. The seminiferous tubules in the right testis were small in diameter, and no spermatogenesis was evident (Fig. 2A, B and C). As a consequence, Sertoli cells with the tubules and Leydig cells within the interstitium appeared prominent. The right epididymis and both ductus deferens were structurally normal but no intraluminal spermatozoa were present (Fig. 2D).

### Genomic and cytogenetic diagnosis

All bulls with intended use for AI in Ireland are screened for karyotype abnormalities using the process outlined in detail by Berry et al. (2017) [11] and Ryan et al. (2024) [8]. The approach uses genotype intensity metrics derived from SNP genotype panels; specifically, the B-allele frequency (BAF) and Log R ratio (LRR). All animals are genotyped for approximately 60,000 SNP genotypes (depending on the version of panel used). The bull in this case was genotyped using the ThermoFisher IDBV6 chip.

**Fig. 3:**
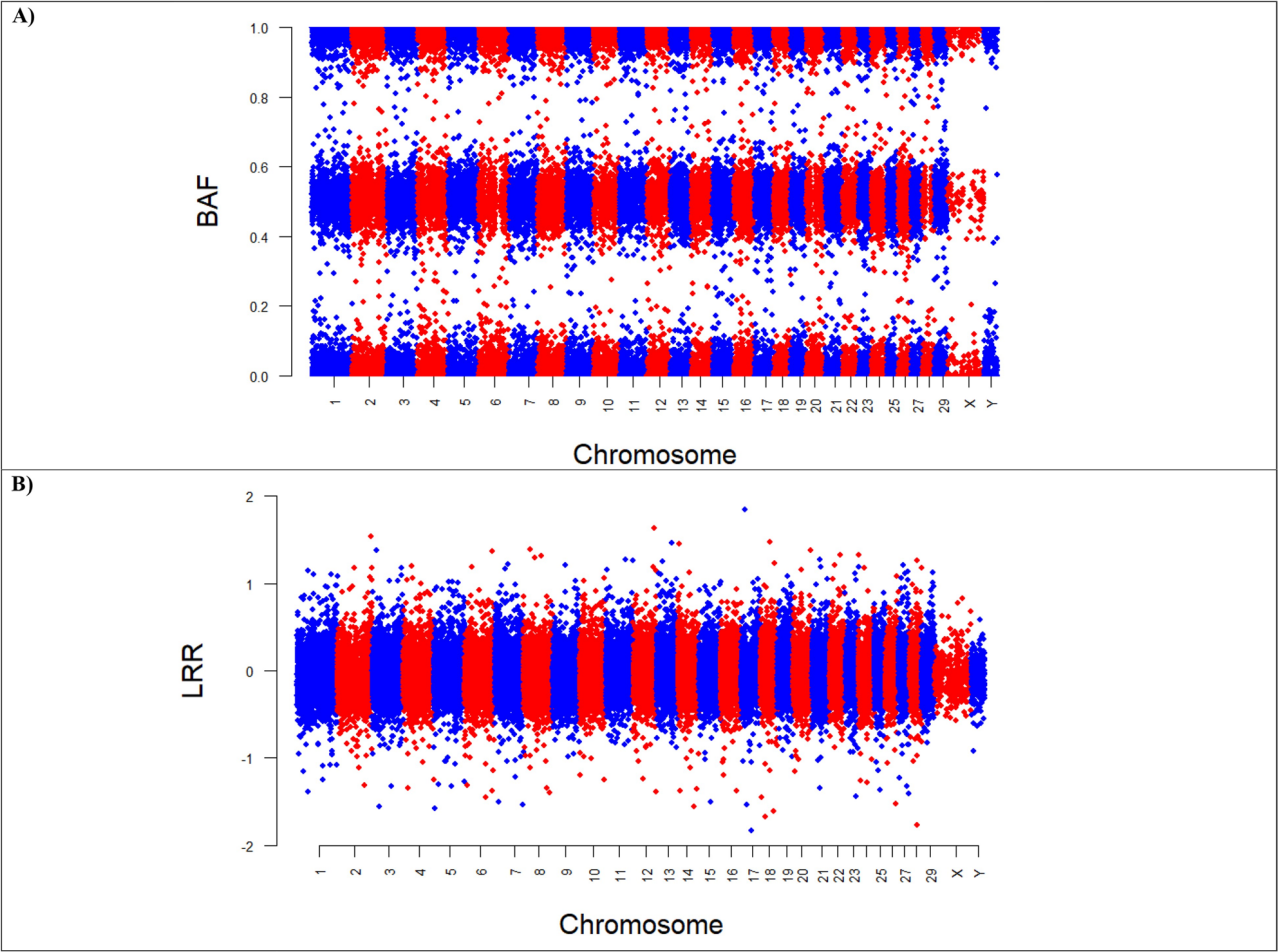
Intensity plots for chromosomes X and Y from a bull with Klinefelter syndrome (61,XXY). **A** B-allele frequency (BAF) and (**B**) Log R Ratio (LRR) whole-genome Manhattan plots for the XXY Klinefelter bull. The X chromosome exhibits a diploid pattern (BAF cluster at 0.5, LRR consistent with autosomes), indicating the presence of two X-chromosomes

Initial aneuploidy screening flagged this bull as potentially XXY based on a high X-chromosome heterozygosity. Specifically, of the 363 non-pseudoautosomal X-chromosome SNP genotypes, 22.3% were heterozygous and 14.5% had a BAF in the diploid range (0.45–0.55), indicating the presence of two X chromosomes (Fig. 3A). This was supported by the standardized X-chromosome LRR being approximately 6 standard deviations above the male mean (Fig. 3B), consistent with the presence of a second X chromosome.

**Fig. 4:**
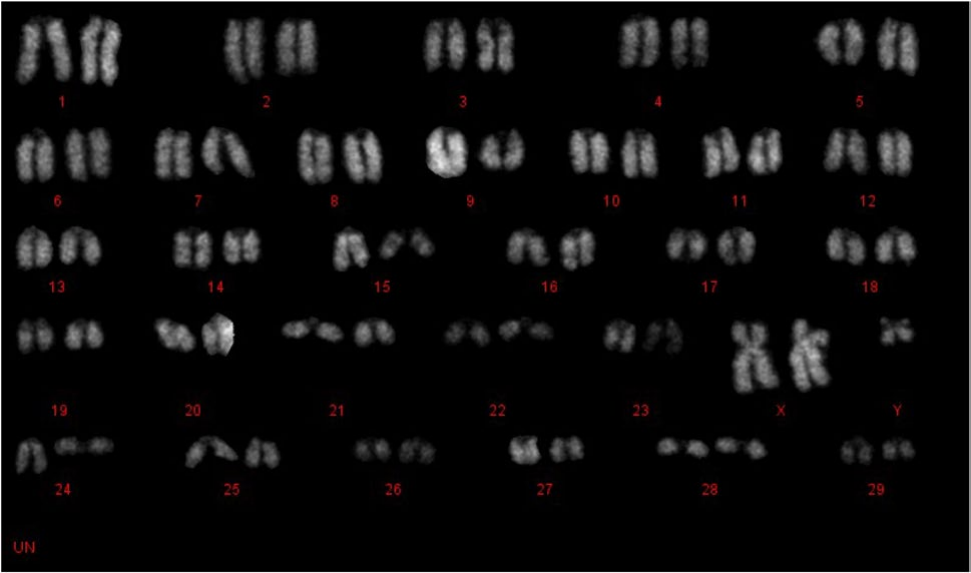
Karyogram of the bull in the case report showing the 61,XXY chromosome complement. Chromosomes are unbanded and arranged by morphology. Two X chromosomes and one Y chromosome are visible in the sex chromosome positions, consistent with the XXY karyotype confirmed by SNP-based genomic analysis

**Fig. 5:**
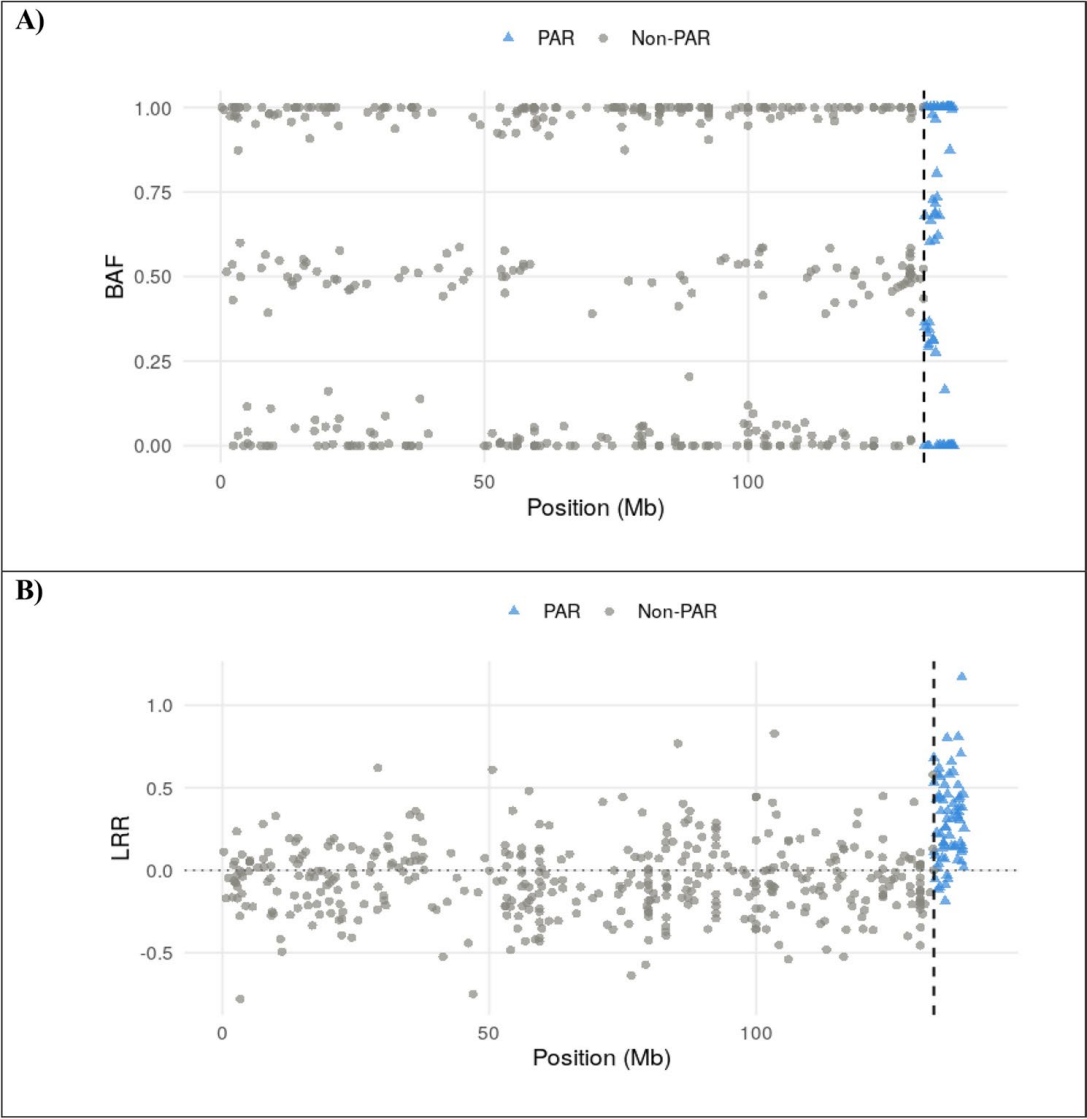
B-allele frequency (BAF, panel **A**) and Log R ratio (LRR, panel **B**) across chromosome X in the 61,XXY case study bull. Blue triangles indicate pseudoautosomal region (PAR) SNPs; grey circles indicate non-PAR SNPs. The dashed vertical line indicates the pseudoautosomal boundary (chrX:133,300,518, ARS-UCD1.2). Elevated LRR and dispersed BAF in the PAR region are consistent with trisomic copy number expected in an XXY individual

As the ThermoFisher algorithm suppresses Y-SNP calls for genotypes predicted as female based on the presence of X-chromosome heterozygosity, the bull had no called Y-chromosome genotypes. Despite this, the standardised Y-chromosome LRR was only 0.9 standard deviations below the male mean, indicating the presence of a Y chromosome. Together with the bull’s male phenotype, this confirmed a preliminary diagnosis of XXY Klinefelter syndrome. Analysis of inheritance patterns from the available parental genotypes confirmed the bull inherited one X-chromosome from the sire and the second X-chromosome from the dam (i.e., XXY due to nondisjunction, with biparental X origin). To further validate the XXY diagnosis, SNPs on chromosome X were classified as pseudoautosomal region (PAR) or non-PAR based on the pseudoautosomal boundary coordinates reported by Johnson et al. [12] for the ARS-UCD1.2 bovine reference assembly. A Hartigan’s dip test for unimodality was applied to the BAF distribution of PAR SNPs using the diptest package in R. Cytogenetic analysis was conducted on the case study using the methodology described in detail by Berry et al. [11]. Cytogenetic analysis was performed on 9 metaphase cells, of which a minimum of 3 were fully karyotyped. The karyogram of the animal is shown in Fig. 4. Analysis of PAR and non-PAR SNPs on chromosome X corroborated the XXY diagnosis. The mean LRR was elevated in the PAR region (+ 0.298) compared with the non-PAR region (-0.057), consistent with the presence of three copies of the PAR in an XXY individual (Fig. 5). The proportion of PAR SNPs with a BAF between 0.25 and 0.75 was 31.3%, compared with 23.0% in the non-PAR region. Hartigan’s dip test strongly rejected unimodality of the PAR BAF distribution (D = 0.1194, *p* < 2.2 × 10⁻¹⁶), indicating a non-unimodal pattern consistent with trisomic copy number in the PAR region.

## Discussion and conclusions

While azoospermia in an XXY bull would ultimately be detected during routine semen evaluation as a young test sire, the underlying cause of the infertility would not be identified without further cytogenetic or genomic investigation. In the field, it may be advisable for clinicians to consider referral to investigate potential chromosomal abnormalities in young bulls presenting with disproportionately small scrotal circumference, azoospermia, or discordance between normal libido and poor semen quality. When an XXY bull is identified through genomic screening, the recommended management decision is immediate removal from the breeding programme without the need for further fertility testing, as the condition is invariably associated with azoospermia and the results from the present study and elsewhere [8, 13] demonstrates that diagnosis can be made with confidence from the genomic data alone. Despite the small stature of the bull presented here, he got to 486 days of age through the rearing units of a busy AI stud dairy breeding program. The results of the Trisomy test were available before the bull attained his AI code, but it is worth noting that costs were incurred by the AI centre to mature this bull over a 1-year production cycle. This test prevented time being wasted in the barns and processing laboratory and this is valuable information to the industry as a whole to flag bulls before resources are channelled into consistent failed attempts at collecting from an infertile bull. Where resources permit, cytogenetic confirmation and post-mortem examination as performed in the present case provide valuable additional information for the scientific community.

Clinical and post-mortem examination of the bull presented here found marked bilateral testicular hypoplasia and an absence of spermatogenesis both of which have been previously described in Klinefelter bovines [6]. The bull had a weight of 380 kg and testicular circumference of 18.5 cm at 480 days of age which is much smaller than the average scrotal circumference of age and breed matched XY bulls (412 +/- 7 kg and 332 +/- 4 mm at 400 days of age [10]). The weight of the vesicular glands was also lower than expected for a bull this age (60–100 g, this bull 32 g) [14]. The bull had a withers height of 135 cm, shorter than that of breed-matched controls (137–152 cm) [15]. The bull in the present case did not have feminine features as reported by Dunn et al. (1980) [6] but was more similar to what had been described by Schmutz et al. (1994) [5], where the bull appeared masculine but smaller in stature. The reduced scrotal circumference and testicular weight too was comparable to other studies on Klinefelter cattle [5], that is, 18 cm in diameter and a combined weight of 80.3 g at 25 months in one report [5] versus 18.5 cm and 89 g in the present report. The smaller scrotal circumference, testicular weight and volume is likely due to the reduction in seminiferous tubule length and diameter which has also been reported by others [9]. Other differentials for smaller scrotal circumference and testicular hypoplasia include testicular degeneration [16], segmental aplasia and persistent Muellerian duct syndrome [17]. However, most of these conditions cause a reduction in sperm production and quality rather than complete azoospermia [17].

The measured testosterone concentration in the present case report indicated that there is Leydig cell endocrine function present [18] and this likely also accounted for normal penile structure and length and the normal accessory sex gland weights in other cases of Klinefelter syndrome in cattle which have been reported by others (5,6 [19]. A single blood sample revealed a testosterone concentration of 13.1 nmol/ml in the present report which was similar to of 12.01 nmol/ml prior to GnRH stimulation, reported by Schmutz et al. (1994) [5]. While there was only one blood sample taken from this animal due to ethical limitations, we still feel that the normal concentration found does help with explaining the normal levels of libido described in the history and also explain how the penis and accessory sex gland developed normally in the present case.

The histopathological finding in the present case were consistent with other reports in the bovine literature [5, 6, 9]. Others [5] have described the histological appearance of the seminiferous tubules as being poorly developed in Klinefelter bovines and partially collapsed with a thick wrinkled basement membrane containing few Sertoli cells and no germinal cells (i.e. Sertoli cell only pattern). Additionally, Schmutz et al. [5] described normal epididymal tissue that is devoid of spermatozoa in a Klinefelter bull. Both hypoplastic seminiferous tubules with a reduced tubular diameter and empty epididymal lumen were noted in the present case report consistent with previous reports. Germ cell loss/‘Sertoli cell only syndrome’ are features of Klinefelter syndrome described in humans in humans (incidence of 1 in 600), with much of the seminiferous tubules being either degenerate or hyalinised with testicular fibrosis in some cases [18, 20].

The azoospermia observed in this and other bovine XXY cases [3, 6] reflects a conserved mechanism documented across mammals. While a single Y-chromosome bearing SRY is sufficient to drive testis development, the extra X chromosome disrupts spermatogenesis through gene dosage imbalance [21]. Although one of the two X chromosomes in XXY individuals is silenced, a proportion of X-linked genes escape this silencing and remain active from both X chromosomes, including genes expressed specifically in the testis, resulting in gene overdosage that impairs the survival of germ cells, the precursor cells that would normally develop into sperm [22, 23]. The precise mechanism driving this germ cell loss remains debated, but the clinical outcome is consistent across species - XXY males in cattle, horses and sheep invariably present with azoospermia despite normal androgen production [3–7].

The incidence of Klinefelter in Irish cattle has been estimated to be 0.0870% in Irish male cattle [8]. Therefore, of the 2,339,250 calves born in Ireland in 2024 (AIM Bovine Statistics Report 2024), approximately half are expected to be male (1,170,000), giving an estimated 1,018 Klinefelter bull calves expected to be born annually, of which the case in the present study was one. Of the 18 Klinefelter male cattle detected by Ryan et al. [8] in an Irish population of 20,670 males, 4 had genotypes available from both parents. Of these four individuals, all had inherited the extra X chromosome from their dam; two inherited one copy of each maternal X-chromosome (i.e., heterodisomy), whereas the other two inherited two copies of the same maternal X-chromosome (i.e., Isodisomy). In contrast, the animal in the present case inherited one X chromosome from each parent, suggesting a non-maternal meiotic error. Thus, whereas the four previously reported Klinefelter males displayed evidence of maternal nondisjunction, the origin of the abnormality in the present case study is consistent with a paternal nondisjunction event arising from paternal meiosis I, as meiosis II errors in the sire would generate XX or YY gametes and cannot give rise to an XXY offspring. Mosaic aneuploidy, specifically XX/XXY mosaicism, cannot be fully excluded on the basis of 9 metaphases alone; however, the consistent BAF and LRR patterns across 485 X-chromosome SNPs, including the elevated PAR LRR and non-unimodal BAF distribution consistent with trisomic copy number in this region (Fig. 5; [4], provide independent whole-genome support for a non-mosaic XXY diagnosis. The most common mechanism underlying paternal XXY nondisjunction is failure of X-Y recombination at the pseudoautosomal region during male meiosis [24]. Thus, the present case study provides the first confirmed evidence of paternal nondisjunction as the origin of Klinefelter syndrome in cattle. By comparison, in humans approximately half of 47,XXY cases have a paternal origin of the extra X chromosome [25]; paternal errors are notably more common in XXY than in autosomal trisomies, where paternal nondisjunction accounts for only approximately 10% of cases [24, 26]. XXY aneuploidy has also been documented in horses [4, 27] and sheep [5] although no parent-of‐origin analyses have been reported in these species.

Klinefelter syndrome represents one of several sex chromosome abnormalities for which early genomic identification offers significant clinical and economic advantages. Prior to the advent of SNP-based genomic screening, all reported bovine XXY cases were identified retrospectively during investigations of infertility, with karyotyping performed only after azoospermia or testicular hypoplasia had been detected [3, 6, 7]. This necessitated that animals reached breeding age before infertility could be diagnosed. In the case of a natural-mating bull, infertility may not be recognised until at least 22 days into the breeding season, with substantial financial repercussions for herd fertility rates [28]. The introduction of national SNP genotyping programmes has fundamentally changed this, enabling identification of XXY animals as calves at the time of routine genotyping, well before any clinical signs are apparent. Karyotyping, while definitive, can be costly [29], making whole-population screening prohibitively expensive; the sample requirements also make it logistically challenging. In contrast, SNP-based aneuploidy screening is performed on data already generated for routine parentage and genomic evaluation, adding no additional cost to existing workflows. In Ireland, approximately 10,000 young bulls destined for AI are genotyped annually, meaning the pipeline described herein screens all potential AI sires for chromosomal abnormalities at no additional cost beyond routine genotyping, a considerable advantage over traditional cytogenetic approaches [29].

The phenotypic presentation of sex chromosome disorders is unpredictable — while most bovine XXY cases present with a normal external male phenotype [3, 6], others have presented with feminine features [7] a completely female phenotype [8], or overtly abnormal genitalia as in XXX/XY chimerism [30]. Clinical appearance alone is therefore an unreliable basis for detection, reinforcing the value of genomic screening for population-level identification of affected animals.

## Conclusion

This case demonstrates a robust framework for diagnosing sex chromosome aneuploidies using routinely available SNP genotype data. Although rare, Klinefelter syndrome should be included in the differential diagnosis of testicular hypoplasia and azoospermia in bulls.

## Data Availability

All data supporting the findings of this study are available within the paper.
